# Genome-Wide Association Study-Guided Exome Rare Variant Burden Analysis Identifies IL1R1 and CD3E as Potential Autoimmunity Risk Genes for Celiac Disease

**DOI:** 10.3389/fped.2022.837957

**Published:** 2022-02-14

**Authors:** Haifa Mansour, Babajan Banaganapalli, Khalidah Khalid Nasser, Jumana Yousuf Al-Aama, Noor Ahmad Shaik, Omar Ibrahim Saadah, Ramu Elango

**Affiliations:** ^1^Department of Genetic Medicine, Faculty of Medicine, King Abdulaziz University, Jeddah, Saudi Arabia; ^2^Princess Al-Jawhara Al-Brahim Center of Excellence in Research of Hereditary Disorders, King Abdulaziz University, Jeddah, Saudi Arabia; ^3^Department of Medical Laboratory Technology, Faculty of Applied Medical Sciences, King Abdulaziz University, Jeddah, Saudi Arabia; ^4^Pediatric Gastroenterology Unit, Department of Pediatrics, Faculty of Medicine, King Abdulaziz University, Jeddah, Saudi Arabia; ^5^Centre of Artificial Intelligence in Precision Medicine, King Abdulaziz University, Jeddah, Saudi Arabia

**Keywords:** celiac disease, auto immune disease, WES, GWAS, PPI, protein modeling

## Abstract

Celiac disease (CeD) is a multifactorial autoimmune enteropathy characterized by the overactivation of the immune system in response to dietary gluten. The molecular etiology of CeD is still not well-understood. Therefore, this study aims to identify potential candidate genes involved in CeD pathogenesis by applying multilayered system biology approaches. Initially, we identified rare coding variants shared between the affected siblings in two rare Arab CeD families by whole-exome sequencing (WES). Then we used the STRING database to construct a protein network of rare variants and genome-wide association study (GWAS) loci to explore their molecular interactions in CeD. Furthermore, the hub genes identified based on network topology parameters were subjected to a series of computational validation analyses like pathway enrichment, gene expression, knockout mouse model, and variant pathogenicity predictions. Our findings have shown the absence of rare variants showing classical Mendelian inheritance in both families. However, interactome analysis of rare WES variants and GWAS loci has identified a total of 11 hub genes. The multidimensional computational analysis of hub genes has prioritized IL1R1 for family A and CD3E for family B as potential genes. These genes were connected to CeD pathogenesis pathways of T-cell selection, cytokine signaling, and adaptive immune response. Future multi-omics studies may uncover the roles of IL1R1 and CD3E in gluten sensitivity. The present investigation lays forth a novel approach integrating next-generation sequencing (NGS) of familial cases, GWAS, and computational analysis for solving the complex genetic architecture of CeD.

## Introduction

Celiac disease (CeD) is an autoimmune gastrointestinal disorder seen in genetically susceptible individuals. The global seroprevalence of CeD (positive for CeD autoantibodies) is estimated to be ~1.4%, while biopsy-proven prevalence is ~0.7% (1, 2). It usually manifests in childhood and early adulthood, but can manifest as early as infancy, necessitating early detection and intervention to prevent irreparable (irreversible) damage like villi atrophy of the small intestine. Diarrhea, abdominal pain, failure to thrive, and anemia caused by intestinal villi atrophy are the most common clinical signs of CeD (3–5). CeD is triggered by abnormal activation of the immune system in response to dietary gliadin, a water-insoluble gluten protein found in wheat, rye, and barley (6, 7). The commonly practiced clinical intervention is adopting a gluten-free diet (GFD); nevertheless, symptoms in some patients persist even after gluten elimination (8, 9). The reliable diagnosis approach for CeD is the histopathological evaluation of small bowel biopsy (SBB), accompanied by the grading of intestinal mucosal lesions based on the pattern of villous atrophy and level of intraepithelial lymphocyte infiltration. Serological testing is a reliable screening approach for detecting tissue transglutaminase (tTG) and endomysial antibodies, but ~5% of celiac patients are seronegative (10).

CeD is a classical multifactorial disease in which an individual's genetic background determines the susceptibility and severity of gluten sensitivity. The strong genetic component implicated in disease etiology has been highlighted in studies conducted among twins, first-degree relatives, animal models, and different ethnic populations (11). A history of biopsy-defined CeD positive family members is expected to account for a greater illness risk in 20% or more of first-degree relatives (2–10-fold) among all the factors indicated [11] Also, patients with autoimmune diseases, such as type 1 diabetes (DM1) (85% are seropositive) (12, 13), primary Sjögren's syndrome, systemic sclerosis, and Graves' disease (autoimmune hyperthyroidism), have an increased chance of developing CeD (14, 15). The environmental factors such as the time of gluten dietary introduction and birth season are also thought to be involved in disease development (16). HLA (HLA-DQA1 and HLA-DQB1) genetic variants encoding the HLA-DQ2 and HLA-DQ8 antigens are known to account (explain) for up to 48% of disease etiology (17). All CeD patients have one of the two risk alleles (90 and 10%), but 30–40% of the general population also carries them (18, 19). This means that HLA risk alleles are simply a prerequisite for the development of CeD.

High-throughput genotyping [genome-wide association study (GWAS)] (20–25), massive parallel sequencing (26–28), and transcriptomics assays (RNA sequencing or microarrays) (29–33) have uncovered numerous genetic variations and differentially expressed genes, providing good resolution into the pathophysiology of CeD in recent decades. However, these studies were largely undertaken in sporadic cases belonging to European/Mediterranean populations (34–37) and were unable to uncover any causative gene underpinning the complicated genetic architecture of CeD. Few whole-exome studies, on the other hand, were able to identify some family-specific rare variants (26, 27). This demonstrates that studying the molecular basis of CeD in families rather than sporadic cases is a promising technique for uncovering novel disease genes or novel variants in known disease genes. However, due to the complicated polygenic nature of CeD, determining a specific causal gene or genetic variant is extremely difficult (27, 28). In this context, exploring the interaction between identified CeD GWAS loci and whole-exome sequencing (WES) variants not only reveal the major heritability but also can aid in uncovering new disease causal genes for many complex diseases (38). This novel approach may also decipher the functional role of some potential loci in any disease.

In recent years, computational integrative annotation of data from GWAS, genome or exome sequencing [next-generation sequencing (NGS)], and genome-wide gene expression data (microarray or RNA seq) have proven to be a powerful approach for interpreting the development and/or progression of several complex autoimmune diseases (39). However, no such integrative genomic annotation studies have been conducted on CeD. Therefore, in the current study, we used WES data from two rare Arab celiac families to develop protein–protein interaction networks between family-specific rare coding variants and GWAS risk loci to unravel the genetic basis of CeD. Our findings demonstrate that even with few rare familial data, applying powerful integrated approaches can help in the identification of potential biomarkers for complex diseases.

## Materials and Methods

**Figure 1:**
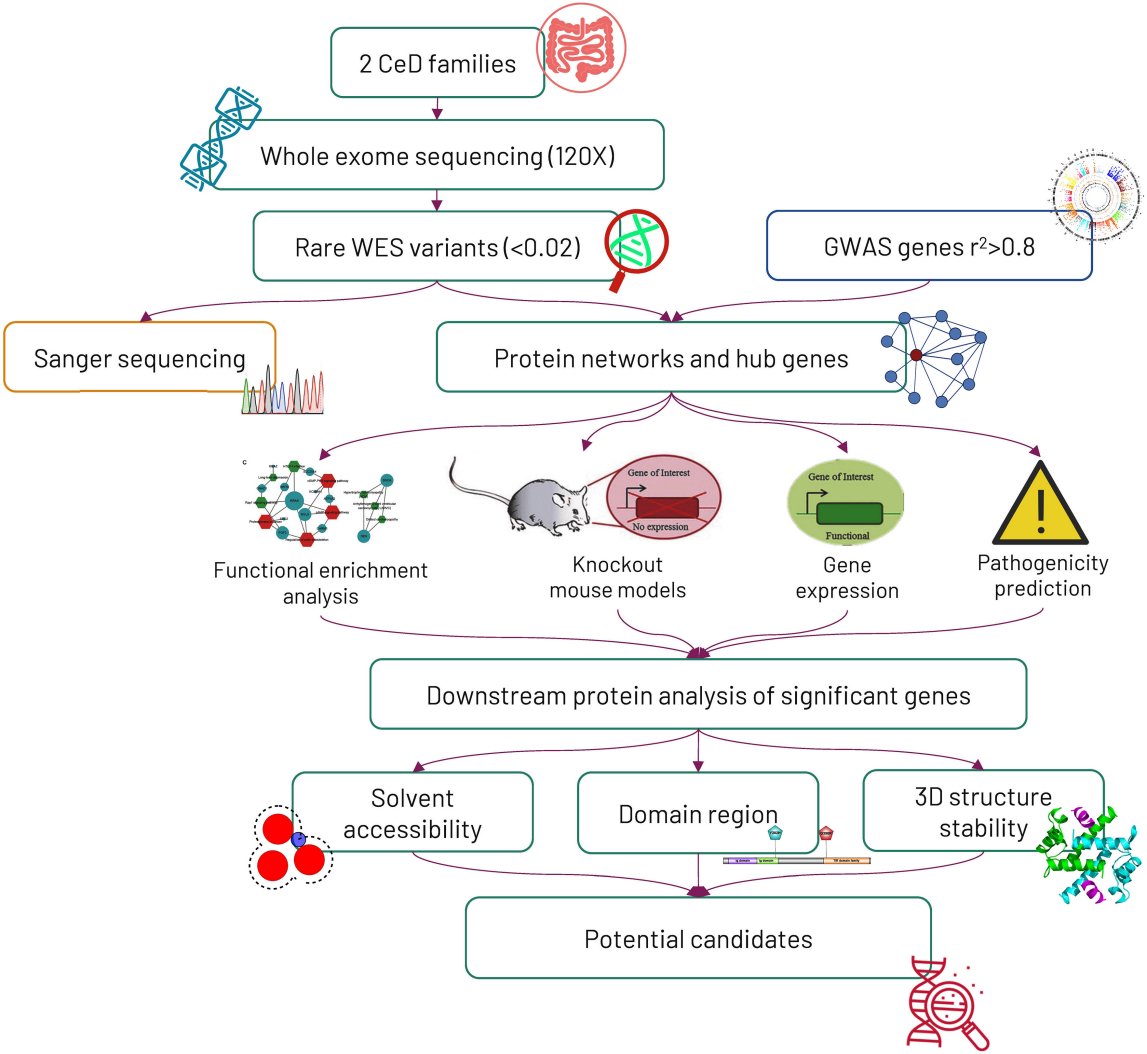
Workflow of the study. Initially, 2 Saudi celiac families were exome sequenced, and later, the mode of variant segregation in the families was determined by the Sanger sequencing method. The rare variants identified from whole-exome sequencing were mapped against genome-wide association study (GWAS) genes, and then the potential hub genes identified from the protein networking were further characterized by computational functional analysis.

The overall study design and experimental approaches are represented in Figure 1.

### Recruitment of Celiac Disease Family

The study protocol was approved by the Research Ethics Committee, King Abdulaziz University Hospital, Jeddah (KAUH). We have recruited two non-consanguineous Arab families living in Saudi Arabia: family A with three affected siblings and family B with two affected siblings. Pediatric gastroenterologists diagnosed the patients by clinical, histopathological (intestinal SBB), and serological (anti-tTG antibodies) examinations. The patients were confirmed to meet the standard diagnostic guidelines of the European Society for Pediatric Gastroenterology Hepatology and Nutrition (ESPGHAN) for CeD (40). A three-generation pedigree of both families was constructed based on personal interviews. Clinical information about the celiac patients was collected from hospital electronic health records. After participant consent was obtained, peripheral blood samples (3–4 ml) were collected and stored at −80°C until genetic analysis was performed.

### DNA Extraction

Genomic DNA was extracted by lysis, binding, elution, and concentration steps outlined in the QIAmp (QIAGEN™, Valencia, CA, USA) blood extraction protocol. DNA concentration and purity were measured at 260 and 280 nm using Nano-Drop 2000 spectrophotometer, respectively, and accepted measurements were 50–150 ng/μl and 1.8–2.0, respectively. The integrity of DNA samples was checked on 1% agarose gel electrophoresis and then stored at −20°C until used for genetic analysis.

### Whole-Exome Sequencing Analysis

WES was performed on the HiSeq2000 Next Generation Sequencer (Illumina, San Diego, CA, USA). The genomic DNA (average of 60 ng/μl) was used for library preparation, including DNA tagmentation (fragmentation and adapter ligation at both ends), target capturing (GKAT), and amplification using the ligated adapters. Libraries then were loaded onto a flow cell and placed on the sequencer for cluster generation and sequencing; the read depth was ~120 ×, covering 97% of target regions (more than 10 ×). The sequencing reads were mapped to the human genome reference GRCH38.p12 assembly using the BWA algorithm, and then SAMTOOLS was used for BAM to SAM files conversion and single-nucleotide polymorphisms (SNPs) and Indel calling (41, 42). ANNOVAR tool was used for rsID identification, annotation, and pathogenicity prediction of variants (43, 44). Variants were filtered based on several quality control (QC) measures like depth (≥30), maximum quality read (≥60), and alternative to total depth ratio (>80% for homozygous variants and 40–70% for heterozygous variants), in addition to other criteria like their minor allele frequency (MAF) (<0.02), location (coding regions), and their pathogenic effects (Supplementary Table 1). All the short-listed variants were analyzed by Sanger sequencing to determine their segregation pattern in the corresponding family members. In this context, oligonucleotide primer sequences (Supplementary Table 2) spanning the variant location were initially designed by Primer NCBI Primer Blast online tool (45), and then standard PCR amplification, Sanger sequencing, sequence alignment, and variant calling steps were performed as described in our recent publications (46, 47).

### Protein–Protein Interaction Networks Construction of Rare Variants Genes and Genome-Wide Association Study Locus Genes

All the WES variants were initially examined to see their mode of inheritance in their corresponding celiac families. Then, we constructed PPINs and examined the interactions between filtered WES genes and CeD GWAS loci [*r*^2^ > 0.8] (20, 21, 48) for families where a classical segregation analysis has failed to identify a single disease causal variant. The WES–GWAS gene list was provided as an input to construct and expand the PPINs by STRING database (https://string-db.org). Cytoscape 3.8.2 software was utilized to view the constructed networks and to calculate the centrality measures (49).

### Network Analysis and Identification of Hub Genes

The PPINs generated from WES–GWAS data of each family were analyzed using two Cytoscape plug-ins, ClueGO (50) and CluePedia (51), for the execution of functional enrichment analysis using Kyoto Encyclopedia of Genes and Genomes (KEGG) pathways and immune system processes as key query Gene Ontology (GO) terms. Furthermore, the degree centrality (DC) parameter of network topology was analyzed utilizing Network analyzer Cytoscape plug-in. DC represents the number of interactions with any nodes in the network (52), and genes with DC > 10 were selected as hub (high-centrality) genes.

### Computational Functional Validation of Selected Potential Celiac Disease Genes

The high-centrality genes from each PPIN were further explored to investigate their potential contribution to disease development. In this context, several databases and computational tools were used to perform functional enrichment annotations, examine gene expression levels in different organs, and note down the altered phenotypes of knockout (KO) mouse models.

#### Gene Ontology Annotations and Pathways

We used the Ensembl database (https://www.ensembl.org/index.html) to analyze the functionally enriched key GO terms including biological processes, molecular function, cellular components, and pathways for all the hub genes.

#### Knockout Mouse Model

In order to gain additional insight into the biological function of each query hub gene, we have used the gene names as the input data in the Mouse Genome Information database (MGI) (http://www.informatics.jax.org) (53). This database provides lists of pathological phenotypes in KO models in reference to the studied mouse strain as well as an overview of the altered phenotypes in the mouse model.

#### Gene Expression Analysis

The gene expression data of the query hub genes were retrieved from the EBI gene expression atlas (EXA) interface available in Ensembl. This tool generates the normalized expression level of each gene in various organs and tissues in the form of a heatmap. Baseline expression level measurements were represented in either fragment per kilobase of exon model per million mapped reads (FPKM) or transcripts per million (TPM).

#### Pathogenic Prediction of Hub Gene Variants

The rare coding variants identified in hub genes were further analyzed by the variant effect predictor (VEP) tool provided by Ensembl (54). From the VEP outputs, prediction scores of SIFT, PolyPhen, CADD, and Mutation assessor were selected. The MAF of these variants was determined by searching in Saudi Human Genome Project (SHGP) (https://shgp.kacst.edu.sa/index.en.html) and Great Middle East (GME) Variome (http://igm.ucsd.edu/gme/) databases.

### Rare Coding Variant Effect on the Protein Structure of Celiac Disease Candidates

The hub genes showing the highest interaction (gene count numbers) with GWAS genes and positive findings from computational annotations were shortlisted and further studied.

#### Protein Structural Feature Analysis

The amino acid sequences in FASTA format were provided as an input to the Protein Families database (http://pfam.xfam.org) for mapping the variants onto functional domains (55). Additionally, the PredictProtein database (https://predictprotein.org) was used to detect the change in solvent accessibility and flexibility of the candidate protein in both native and variant conditions.

#### 3D Structure Stability Analysis

Homology protein modeling of the query proteins was performed using BLASTP (56) and Swiss PDB viewer (https://swissmodel.expasy.org) tool by searching for the experimentally solved structures (with >50% coverage) deposited in protein data bank (PDB) (https://www.rcsb.org) (57). After that, the Modeler 10.1 software was utilized to build the protein model using the multiple template alignment approach. A total of 100 models for each protein were initially built, and then the models with the lowest DOPE scores were further selected to perform energy minimization of the 3D structures built (58). The optimum 3D structure was validated using the Ramachandran plot in the PROCHECK program (59), which was eventually used as a reference to build the mutant protein version with the DUET webserver. Besides predicting the tertiary structure models, DUET also provides the consensual stability scores of SDM (assess the change in amino acids function and protein family) and the mCSM (assess missense mutation effect of the protein structure) methods (60). Finally, Pymol software was used for visualization and alignment of all the protein structures built (61).

## Results

### Clinical and Family History

**Figure 2:**
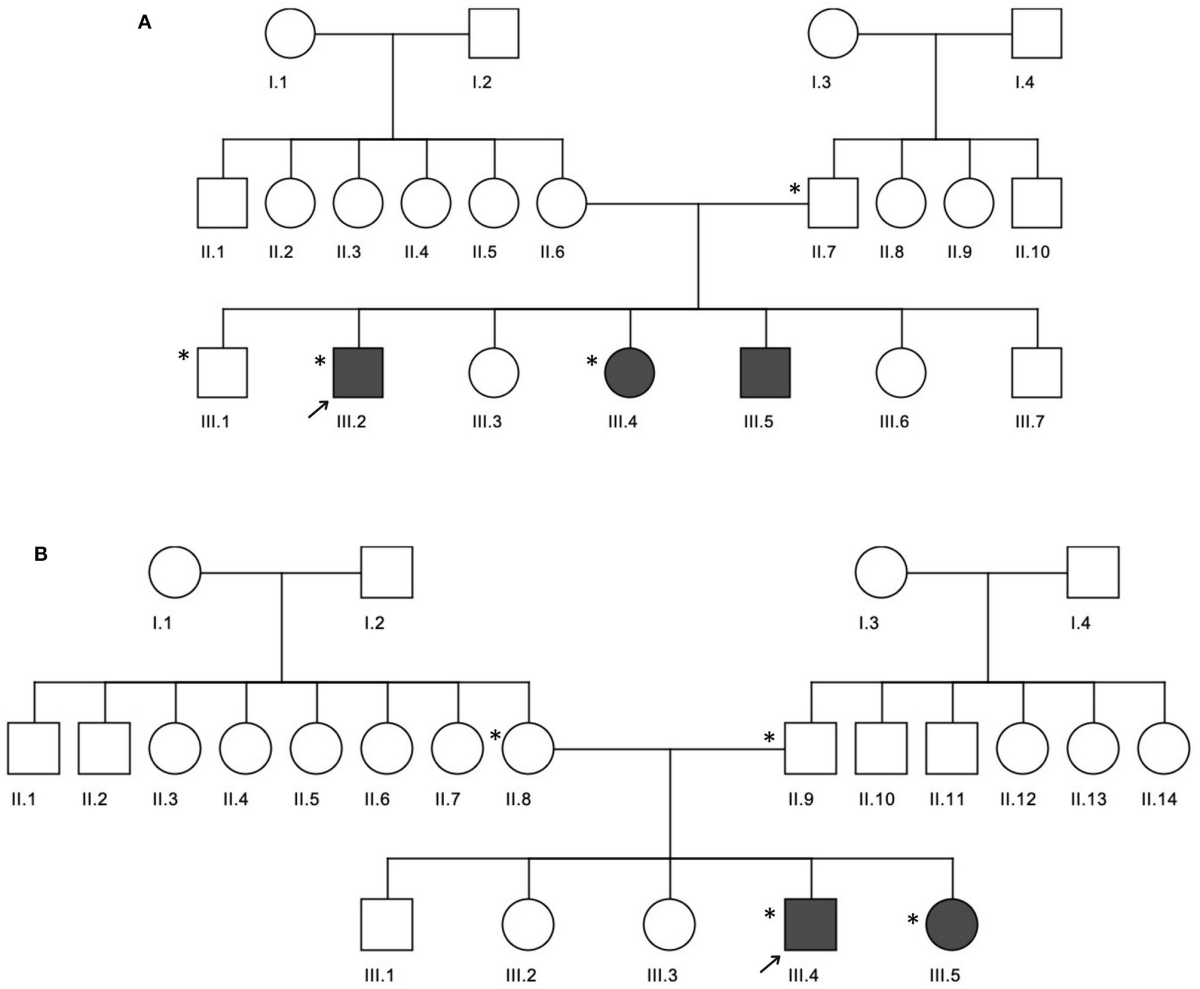
Three generation pedigrees of celiac disease families. **(A,B)** The pedigrees for families A and B, respectively. The gray circle or boxes represent patients with celiac disease. Exome-sequenced individuals are indicated by the * symbol.

In family A (Figure 2A), the age of CeD diagnosis for the proband and two siblings are 18 years for III.2 and 12 years for III.4 and III.5, and the latter two showed elevated levels of tTG antibodies on an average level of 234.7 chemiluminescence unit (CU) when the normal range is <20. All the 3 patients adopted a GFD after 1 month of histological diagnosis, in addition to several nutritional supplementations like calcium, magnesium, zinc, Vit D, iron, and folic acid to compensate for the metabolic defects of CeD. Proband (III.2) was prescribed thyroxine tablets to manage the high level of thyroid-stimulating hormone (TSH) and hypothyroidism.

In family B (Figure 2B), the age of diagnosis was 5 years for III.4 and years for III.5 with a 7 CU average of tTG antibodies. Similar to family A, both patients adopted a GFD after 1 month of histological diagnosis and several nutritional supplementations like calcium and Vit D, in addition to antihistamine and pain killer drugs. Patient III.4 is diagnosed with diabetes mellitus; therefore, he was prescribed insulin, as well as thyroxine tablets for hypothyroidism management.

### Whole-Exome Sequence Analysis

An average of 75,815 and 104,377 variants (with a Phred quality score of Q30) were identified in families A and B, respectively. In family A, a total of 338 variants (27 homozygous and 311 heterozygous) spanning over 322 genes were shared among III.2 and III.4. In family B, III.4 and III.5 shared 313 variants (37 homozygous and 276 heterozygous) mapped to 271 genes. The majority (11/12; 91.6%) of the coding variants identified in both families belonged to the missense category. Supplementary Table 3 shows the WES variant filtration steps followed in this study.

### Segregation Analysis

Sanger sequencing validation of potential variants was performed to determine their mode of inheritance, i.e., autosomal recessive (AR), compound heterozygous (CH), or *de novo* (DN), in the CeD families. Overall, WES data filtration under different combinations yielded 4 variants under the AR mode of inheritance. These variants include IGFN1, c.3056T>G and LAD1, and c.452G>A variants for family A; and SSPO, c.11582dupA, PKD1L2, and c.706_707delAA variants for family B. However, Sanger sequencing did not confirm the AR segregation of the potential variants (IGFN1, c.3056T>G and LAD1, and c.452G>A) in the individuals from family A (Supplementary Figures 1, 2). Of the candidate variants short-listed for family B, SSPO was reported to be a pseudogene, and PKD1L2 has no functional correlation with CeD. Moreover, the search for CH variants in family A was not possible due to the absence of maternal WES data. In the case of family B, two genes with multiple variants showing CH inheritance were found, CYP4X1, c.116C>T (maternal), c.377C>T (paternal), and FLG, c.4765C>T (maternal), and c.6001G>A (paternal). However, they were excluded due to the lack of functional relevance to autoimmunity and CeD. Therefore, it is concluded that both AR and CH segregation models cannot explain the genetic basis of CeD in these two families.

### Protein–Protein Interaction Network Construction and Expansion

The segregation analysis of the rare coding variants did not provide any evidence of causal gene(s) for CeD. So we hypothesized that the enrichment of variants in many functionally related or interacting genes in a relevant pathway might provide a clue to the disease biology. In families A and B, we identified rare variants in 322 and 271 genes, respectively. We found 23 and 13 of these genes from families A and B, respectively, in the innate immunity database (Supplementary Table 4). We constructed PPINs with genes (322 and 271 genes) from WES data and CeD GWAS loci [50, *r*^2^ > 0.8].

Table 1 shows the statistical parameters of WES–GWAS PPINs before and after their expansion using the STRING database. In the case of the WES results from family A, only BACH2 (a GWAS gene) had one copy of the missense variant (rs1321699864), and it was not found to interact with any other WES identified genes, while in family B, no GWAS genes were found to have any rare coding variants. The maximum PPIN enrichment *p*-value was 9.99 × 10^−16^, and the minimum average local clustering coefficient was >0.423. Fine mapping of CeD GWAS loci on the immunochip platform has concluded 57 loci mapped to 50 genes with linkage disequilibrium score (*r*^2^) of >0.8 (20, 21, 48). The PPIN mapping and expansion of 371 genes (50 GWAS and 322 WES genes) in the STRING database have shown the direct interactions between 42 GWAS (84%) and 65 WES (20.1%) genes in family A. For family B, 321 genes (50 GWAS and 271 WES genes) were mapped and expanded showing direct protein–protein interaction between 44 (88%) GWAS and 56 (20.6%) WES genes.

**Table 1:** Statistical parameters of original and expanded WES–GWAS protein networks generated by STRING database.

**Statistical measure**	**Family A**		**Family B**	
	**Before**	**After**	**Before**	**After**
	**expansion**	**expansion**	**expansion**	**expansion**
No. of mapped nodes	359	409	317	357
No. of edges	481	972	423	824
Average node degree	2.68	4.75	2.67	4.62
Avg. local clustering coefficient	0.363	0.392	0.388	0.423
Expected number of edges	355	745	280	583
PPI enrichment *p*-value	1.31 × 10^−10^	9.99 × 10^−16^	11.1 × 10^−15^	<1.0 × 10^−16^

*WES, whole-exome sequencing; GWAS, genome-wide association study; PPI, protein–protein interaction*.

### Functional Enrichment Analysis of Whole-Exome Sequencing–Genome-Wide Association Study PPINs

The functional enrichment analysis of WES–GWAS protein networks has confirmed the predominant role of immune system-related GO terms and pathways in CeD etiology. In family A, 4.9% of WES and 20% of GWAS genes belonged to immune-related pathways, when compared with the total direct interactions. These include regulation of innate and adaptive immune responses, cytokine–cytokine receptor interaction, and regulation of production of molecular mediators of immune response pathways. On the other hand, 16.6% of the WES genes identified in family B were interacting with 48% of GWAS genes in immune pathway interactions. These genes were associated with autoimmune diseases like DM1, inflammatory bowel disease, rheumatoid arthritis, systemic lupus erythematous, and autoimmune thyroid disease and mapped to the intestinal immune network for IgA production, regulation of innate and adaptive immune response, and B cell- and T cell-mediated immunity pathways.

### Protein Interaction Centrality Measures and Hub Gene Identification

**Figure 3:**
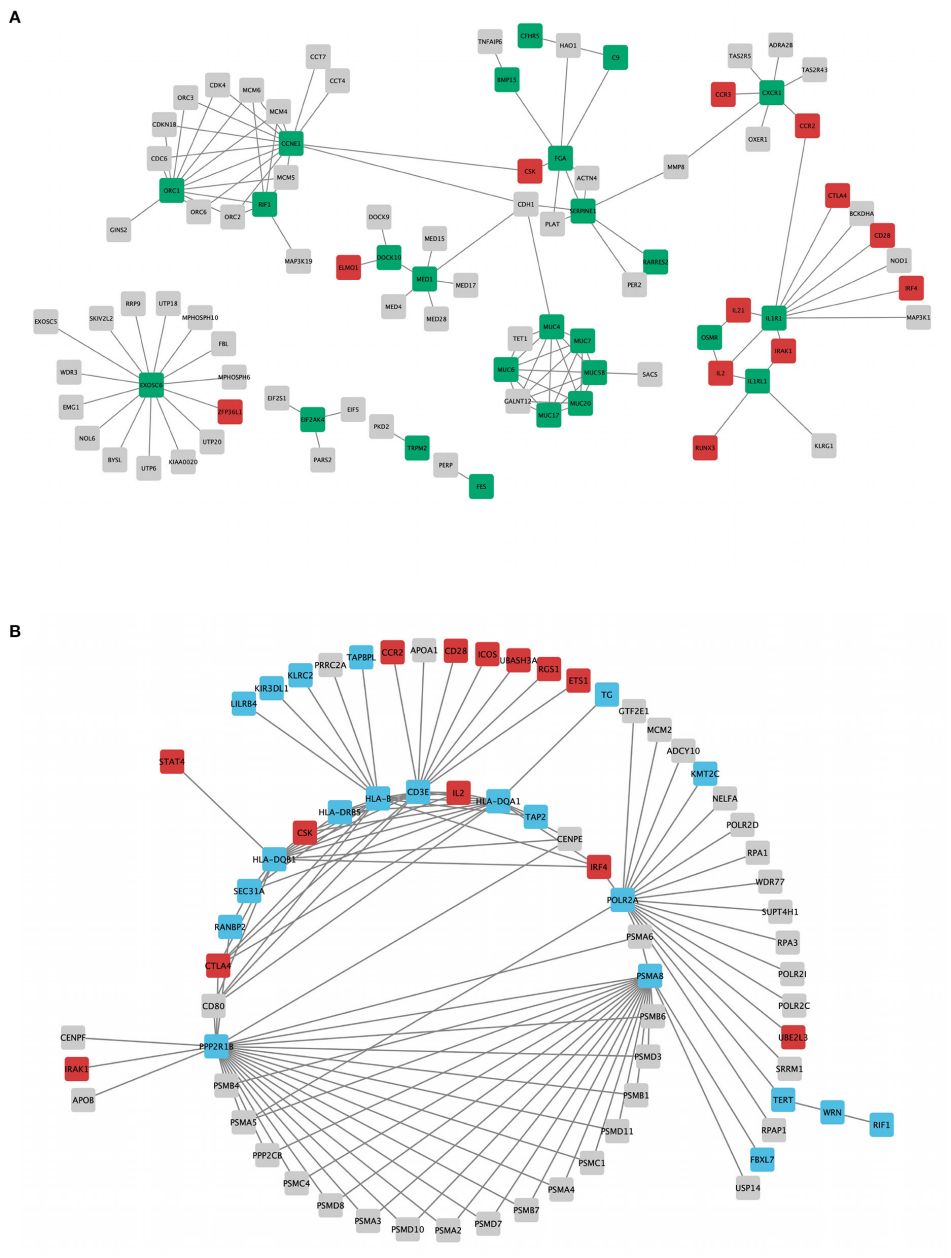
Rare variant mapped genes and genome-wide association study (GWAS) locus gene protein interaction sub-networks of families A **(A)** and B **(B)**. Green (family A) and blue (family B) nodes represent whole-exome sequencing (WES)-identified genes, while red nodes represent GWAS-identified loci with *r*^2^ > 0.8, visualized using Cytoscape 3.8.2 software.

The topology parameters of both PPINs revealed a total of 11 non-HLA WES genes showing a high-centrality score (>10 nodes). HLA genes were excluded to prioritize non-HLA immune-related genes and to study their relevance to CeD. In family A, 4 hub genes—EXOSC6 (Pro272Ser), CCNE1 (Asn260lle), ORC1 (Met816Thr), and IL1R1 (Tyr202His and Gly398Arg)—were identified. In family B, seven hub genes, namely, PPP2R1B (Arg549Cys), FBXL7 (Thr292Ile), PSMA8 (Val11Leu), POLR2A (Lys1838fs), CD3E (Ala157Val), WRN (Thr324Ala), and RANBP2 (Ile664Val), were identified. Of the 11 hub genes, CD3E and IL1R1 have shown the highest number of interactions with GWAS genes, with 9 and 7, respectively. Table 2 and Figure 3 represent the hub genes based on their DC and interacting gene partners from GWAS and WES data.

**Table 2:** Degree centrality between hub genes with GWAS loci and WES mapped genes with rare variants.

**Family**	**Gene**	**Degree of centrality**	**GWAS genes**	**WES genes**
Family A	EXOSC6	15	ZFP36L1	NOL6, WDR3, KIAA0020, EMG1
	CCNE1	14	CSK	ORC1, CCT4
	ORC1	12	–	RIF1, CCNE1
	IL1R1	10	CCR2, CD28, CTLA4, IL2, IL21, IRAK1, IRF4	NOD1, MAP3K1, BCKDHA
Family B	PPP2R1B	25	CTLA4, IRAK1	APOB, CENPF
	FBXL7	22	–	GEMIN5, PSMA8, LRFN3, ANKRD9, MIB2
	PSMA8	20	–	FBXL7, PPP2R1B
	POLR2A	19	IRF4, UBE2L3	WDR77, ADCY10, KMT2C, NELFA, RPAP1, TERT, SRRM1
	CD3E	15	CSK, IL2, CTLA4, UBASH3A, ICOS, ETS1, RGS1, CCR2, CD28	HLA-DQA1, HLA-DRB5, HLA-B, HLA-DQB1
	WRN	11	RMI2	ASCC3, BIVM-ERCC5, BOD1L1, RIF1, TERT
	RANBP2	10	ZMIZ1, UBE2L3	ZNF44, SEC31A, SRRM1, PPP2R1B, CENPF, GEMIN5

*WES, whole-exome sequencing; GWAS, genome-wide association study*.

### Computational Functional Validation of Identified Hub Genes

#### Gene Ontology Annotations and Pathways

**Figure 4:**
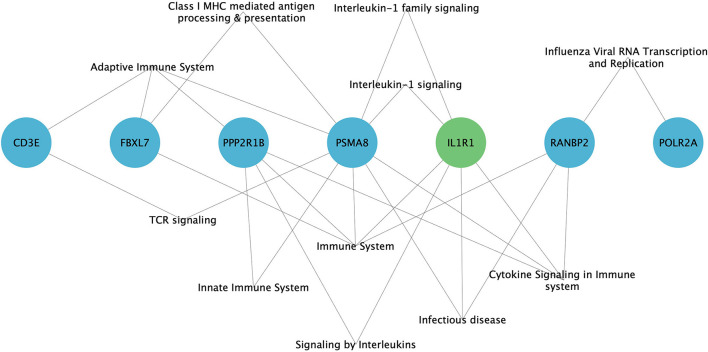
Enrichment of immune system-related pathways in hub genes of family A (green) and family B (blue), visualized using Cytoscape 3.8.2 software.

GO analysis has revealed the enrichment of 7/11 (63.6%) hub genes against immune system-related pathways and annotation terms (Figure 4 and Supplementary Table 5). The adaptive immune response pathway term was enriched for 4 hub genes (CD3E, FBXL7, PSMA8, and PPP2R1B). Only two genes (PSMA8 and PPP2R1B) were seen playing a role in the innate immune response pathway. IL1R1, PPP2R1B, and PSMA8 were involved in the interleukin signaling (IL-1, IL-10, and IL-17) pathway. RANBP2 was the only hub gene found to be connected to the interferon signaling pathway. GO enrichment annotations have shown the involvement of CD3E in T-cell selection, differentiation, activation, T-cell receptor complex binding, and signaling and inflammatory response and inflammatory response regulation for IL1R1.

#### Knockout Mouse Model

**Figure 5:**
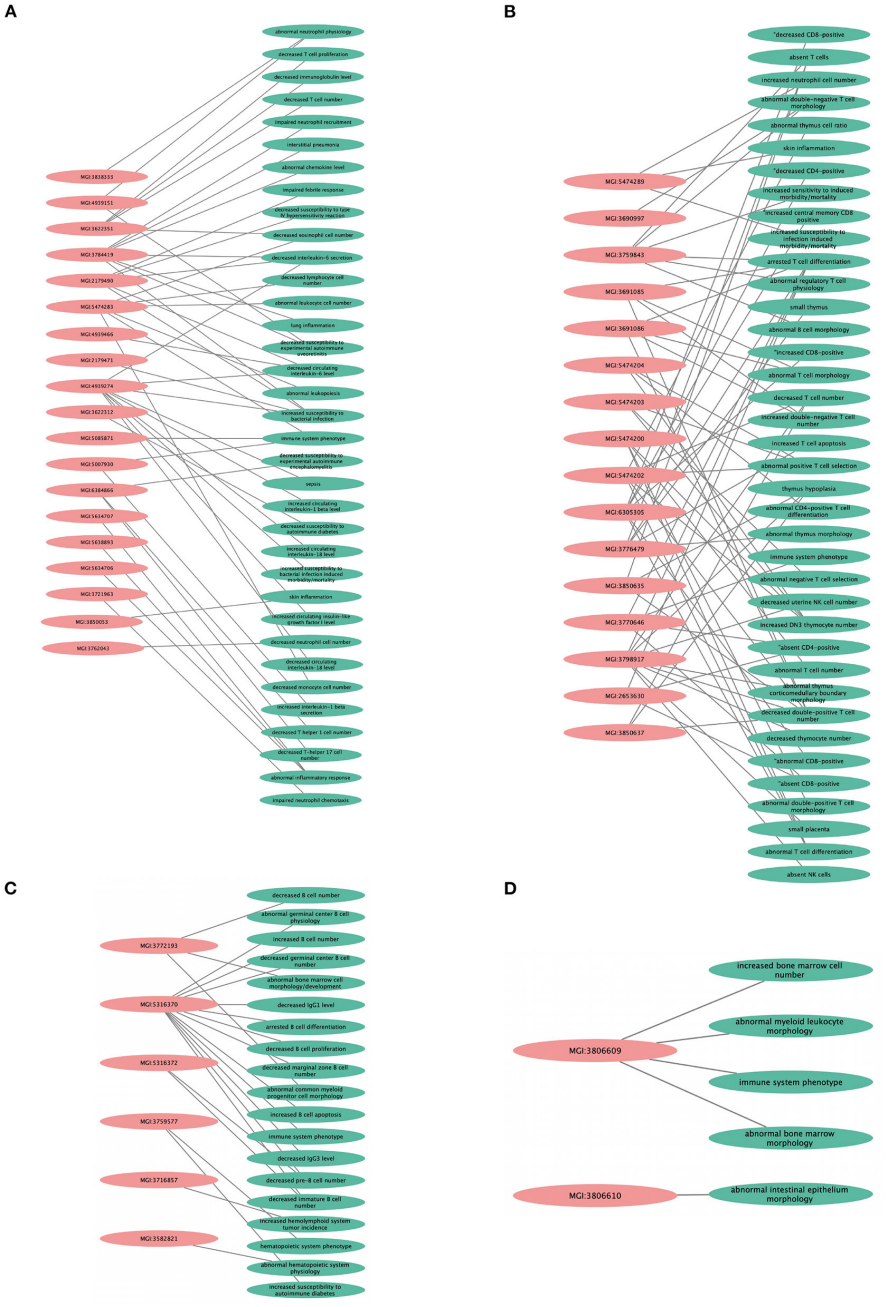
Knockout mouse phenotype model analysis result from Mouse Genome Information (MGI) database for the selected genes, like **(A)** IL1R1, **(B)** CD3E, **(C)** POLR2A, and **(D)** CCNE1. The figure represents immune-related altered phenotypes (green) with the corresponding mice genotype ID (pink), visualized using Cytoscape 3.8.2 software.

We found that the KO models of 4 (36.3%) of the 11 hub genes have demonstrated altered immune system phenotypes (Figure 5). The CD3E KO mouse showed defective functional phenotype related to T cells (selection, differentiation, morphology, and number) and the thymus (morphology, size, cell ratio, and number). The IL1R1 KO mouse models showed leukocyte count changes including lymphocytes (T cells, B cells, and natural killer cells), granulocytes (eosinophils and neutrophils), and monocytes, as well as abnormal circulating interleukins levels including IL-1, IL-6, and IL-18. POLR2A KO mouse displayed abnormal B cell count, increased apoptosis, decreased proliferation, arrested differentiation, and increased susceptibility to DM1. Abnormal bone marrow and intestinal morphology were also observed in POLR2A and CCNE1 KO models.

#### Gene Expression

**Figure 6:**
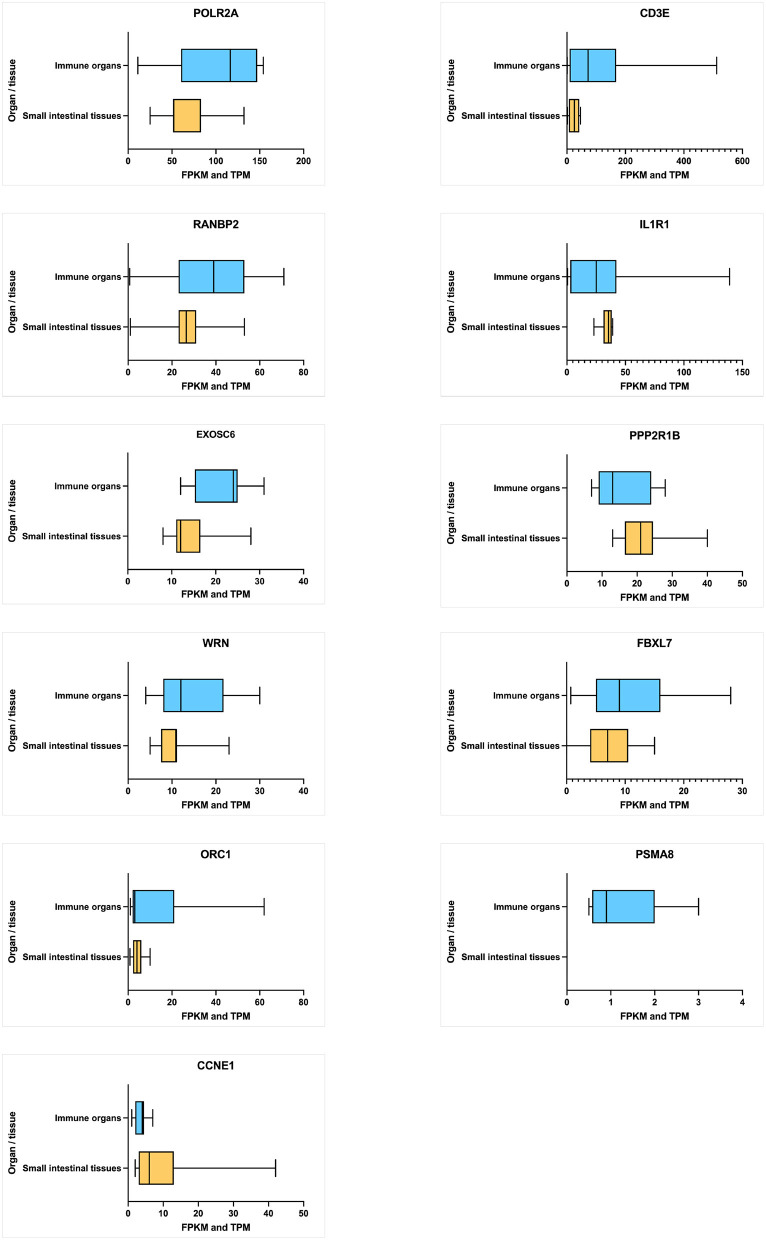
Average level of hub gene expression reported in transcriptomic gene expression databases provided by Ensembl. Small intestinal tissues include the small intestine and duodenum tissue. Immune organs include the leukocyte, spleen, lymph node, thymus, Epstein–Barr virus (EBV)-transformed lymphocyte, bone marrow, and small intestine Peyer's patch. Gene transcription levels are represented in TPM (transcripts per million). Transcription scale: low (0–10), medium (11–1000), and high (>1,000). The figure is generated using GraphPad Prism 9.2.0 software.

The average expression level of each hub gene in small intestinal tissues (small intestine and duodenum) and immune function-related organs (leukocyte, spleen, lymph node, thymus, Epstein–Barr virus (EBV)-transformed lymphocyte, bone marrow, and small intestinal Peyer's patches) (Figure 6) were estimated. Under the immune organs category, the genes, which showed the highest to lowest level expression measured in terms of transcripts per million (TPM), are CD3E (126.5 TPM), POLR2A (101.7 TPM), RANBP2 (34.8 TPM), and IL1R1 (30.1 TPM), whereas in the case of small intestinal tissues, POLR2A has shown the highest expression level of 71.7 TPM, followed by RANBP2 (27.0 TPM), PPP2R1B (24.22 TPM), IL1R1 (21.88 TPM), and CD3E (18.5 TPM) genes.

#### Pathogenicity Characterization of Variants in Hub Genes

We performed the pathogenicity characterization of missense variants identified in the hub genes. In family A, five heterozygous rare variants (MAF ≤ 0.014) spanning 4 hub genes (IL1R1, CCNE1, EXOSC6, and ORC1) were identified (Table 3). But in family B, 7 rare (MAF < 0.016) variants were found in 7 hub genes (CD3E, FBXL7, POLR2A, PPP2R1B, PSMA8, RANBP2, and WRN) (Table 3). Computational predictions confirmed that missense variants of CCNE1, IL1R1, PPP2R1B, PSMA8, and CD3E are deleterious to the function of corresponding proteins (>0.5) (Table 3).

**Table 3:** Rare coding variants of the hub genes from WES–GWAS protein networks of families A and B.

**Gene name**	**Variants from WES analysis**					**MAF**			**VEP**			
	**Chr. no**.	**rs ID**	**cDNA position**	**Amino acid position**	**Effect**	**gnomAD**	**SHGP**	**GME**	**SIFT**	**PolyPhen**	**CADD**	**Mutation assessor**
**Family A**												
EXOSC6	16	rs149061783	c.814C>T	p.Pro272Ser	Missense variant	0.005	0.002	0.001	0	0	13	0.019
CCNE1	19	rs61750863	c.779A>T	p.Asn260lle	Missense variant	0.002	0.003	0.002	0.03^*^	0.767^*^	25	0.641^*^
ORC1	1	rs34521609	c.2447T>C	Met816Thr	Missense variant	0.015	0.007	0.011	0.14	0.05	18	0.033
IL1R1	2	rs34889382	c.604T>C	p.Tyr202His	Missense variant	0.002	0.014	0.009	0.29	0.031	10	0.098
IL1R1	2	rs34835752	c.1192G>A	Gly398Arg	Missense variant	0.002	0.014	0.008	0.36	0.285	16	0.593^*^
**Family B**												
PPP2R1B	11	rs566998075	c.1645C>T	p.Arg549Cys	Missense variant	0.000	0.022	0.011	0^*^	1^*^	32	0.96^*^
FBXL7	5	rs202118294	c.875C>T	p.Thr292Ile	Missense variant	0.002	0.005	0.004	0.72	0.061	20	0.111
PSMA8	18	rs550786252	c.31G>C	p.Val11Leu	Missense variant	0.001	–	–	0.02	0.59	24	–
POLR2A	17	rs1490940612	c.5511_ 5512delCA	p.Lys1838fs	Frame shift variant & splice region variant	–	–	–	–	–	–	–
CD3E	11	rs140639753	c.470C>T	p.Ala157Val	Missense variant	0.000	0.001	0.001	0.02^*^	0.971^*^	25	0.839^*^
WRN	8	rs1800390	c.970A>G	p.Thr324Ala	Missense variant	0.003	0.015	0.016	0.5	0.003	0	0.082
RANBP2	2	rs746730990	c.1990A>G	p.Ile664Val	Missense variant	0.000	–	–	0.61	0	11	0.298

*GWAS, genome-wide association study; WES, whole-exome sequencing; MAF, minor allele frequency; VEP, variant effect predictor*.

* *Significant VEP score*.

### Rare Coding Variants Effect on the Protein Structure of Celiac Disease Candidates

**Figure 7:**
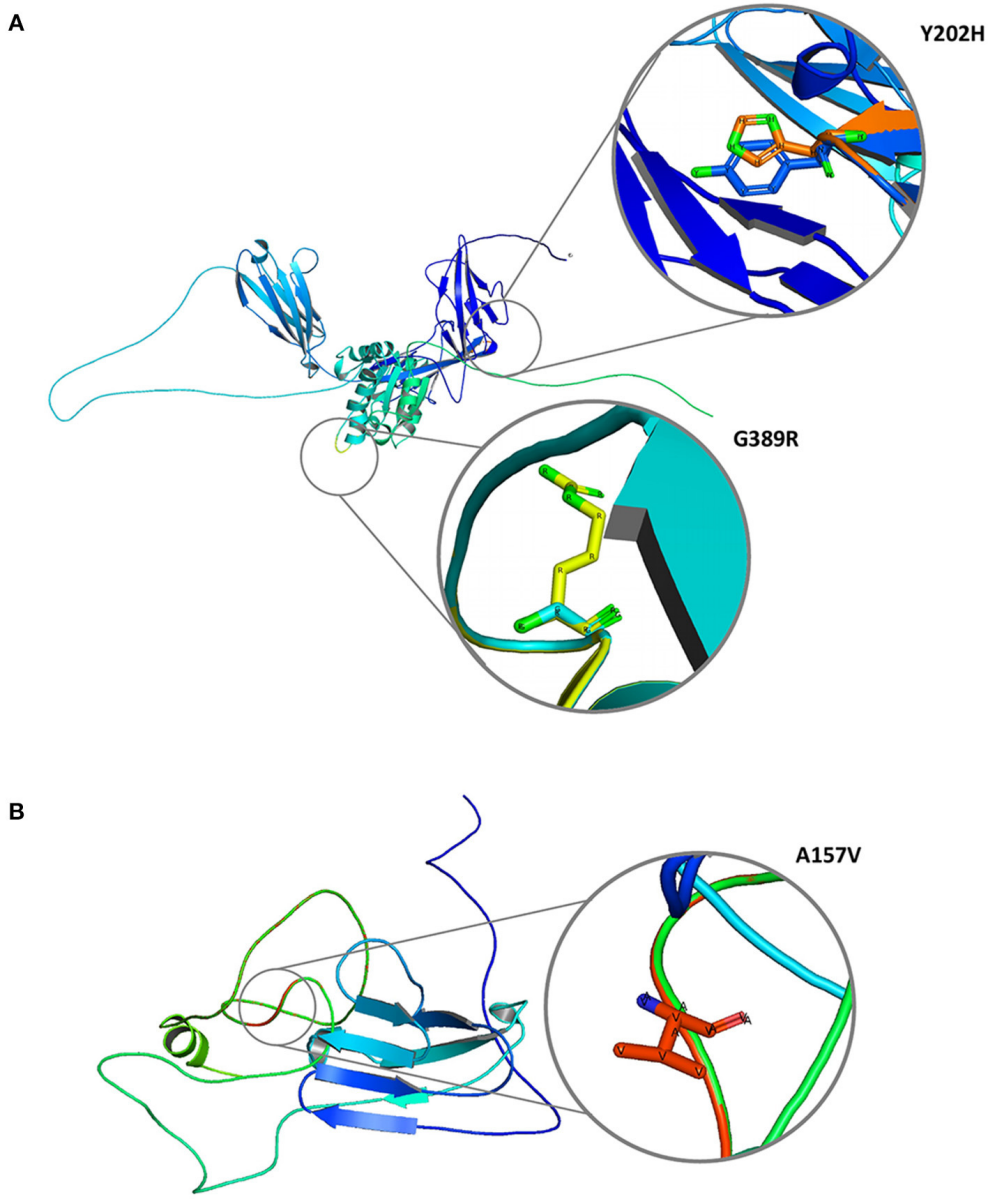
Molecular graphical visualization of normal and mutated 3D protein structure models using Pymol software. **(A)** IL1R1 protein variants Y202H and G389R. **(B)** CD3E protein variant A157V.

The 2/11 (18%) hub genes (IL1R1 and CD3E) prioritized from network analysis owing to their highest interaction with GWAS genes were further explored by structural annotations. Accordingly, the variants identified in the above 2 genes were studied by protein domain mapping, stability, residue flexibility, and solvent accessibility methods (Figures 6, 7).

#### Protein Structure Feature Analysis

The Y202H and G398R variants of IL1R1 gene are localized in the immunoglobulin domain and Toll-interleukin receptor domain regions, respectively. Solvent accessibility findings suggest that the Y202H variant lowers the residue access to solvents, while the G398R variant showed no effect. However, G398R increased the relative B-value (PROFbval), causing the residue to be more flexible. In contrast, the CD3E (A157V) variant was mapped 30 amino acids downstream to Ig-like domain on T-cell surface glycoprotein CD3 epsilon chain but did not influence the residue solvent accessibility. However, this variant is seen to decrease the PROFbval, resulting in lower residue flexibility (62).

#### 3D Structure Stability Analysis

The candidate protein constructs (native) showing the lowest DOPE score were selected for determining the variant effects on protein stability; Ramachandran computational validation plots are represented in Supplementary Figure 4. All 3 variants have shown negative delta values of folding free energy upon mutation (average of −0.9) in kcal/mol measured by mCSM, SDM, and DUET. This shows a destabilizing effect on the protein structure as per consensus predictions shown in Table 4.

**Table 4:** Protein sequence annotation and structural stability prediction results for IL1R1 and CD3E variants.

	**Gene**	**IL1R1**		**CD3E**
	Variant	Y202H	G398R	A157V
Protein sequence annotations	Solvent accessibility	Decreased	No effect	No effect
	Domain regions	1	1	None
	B-value	No effect	Higher	Lower
Protein structure stability prediction	mCSM	−1.425 kcal/mol (*destabilizing*)	−0.068 kcal/mol (*destabilizing*)	−0.629 kcal/mol (*destabilizing*)
	SDM	−1.14 kcal/mol (*destabilizing*)	−2.39 kcal/mol (*destabilizing*)	−0.39 kcal/mol (*destabilizing*)
	DUET	−1.34 kcal/mol (*destabilizing*)	−0.311 kcal/mol (*destabilizing*)	−0.415 kcal/mol (*destabilizing*)

## Discussion

The genetic heterogeneity of CeD cannot be explained by classical genetic segregation methods, as the single gene model is unable to dissect the disease's molecular aspects. Several rare variant burden (RVB) analyses from large-scale WES have been successful in understanding the molecular basis of a broad range of complex diseases including epilepsy (63), autism (64), schizophrenia (65), and amyotrophic lateral sclerosis (ALS) (66). A recent study had proven the power of combining WES and GWAS on a wide level to study multiple complex diseases (67). Nonetheless, our study has more targeted findings, as we selected rare familial cases to benefit from the shared genetic composition in the affected siblings (nonetheless, since we selected rare familial cases to benefit from the shared genetic composition of the affected siblings, our study had more focused findings). Although the two families had distinct genetic findings, the RVB–GWAS approach has helped us identify at least three potential major contributors in these familial cases.

Overall, our analysis identified several possible candidates, and based on the functional enrichment analysis, we prioritized IL1R1 in family A and CD3E in family B. The enrichment analysis of the interleukin 1 receptor type I (IL1R1) gene revealed its involvement (along with many GWAS genes) in T helper 1 immune response, which is the first stage of gluten peptide recognition by HLA antigen. Another major pathway is cytokine signaling, specifically IL-1 and IL-10. The ligands IL-1α and IL-1β bind to IL1R1 to form the IL1R complex with the involvement of other receptors. Both ligands are members of IL-1 family cytokines, a group of signaling molecules involved in innate and adaptive immune response as well as inflammatory processes (68). IL-1β is a key cytokine involved in innate immune response, while IL-1α is mainly found in epithelial and mesenchymal cells in apoptotic and inflammatory conditions (69). High levels of IL1R1 ligands have been linked to elevated tTG IgA serum levels and a higher CeD risk (70–72). Patients who followed a GFD, on the other hand, had lower levels of these ligands (70). Furthermore, an *in vitro* study reported an intense production of IL1R1 ligands by peripheral monocytes in response to pepsin digested gliadin with the involvement of several innate immune system pathways (73). The IL-1 system especially IL-1β is also thought to initiate and regulate IL-23 production, which has been involved in tissue-specific autoimmunity (74).

CD3E, which codes for the epsilon subunits of the cluster of differentiation 3 (CD3) T cell co-receptor, is another CeD candidate. TCR/CD3 complex signaling is essential for the antigen-specific T-cell response as a part of the adaptive immune response to external pathogens, self-antigen, transplanted tissue or organ, and the gluten peptide in the CeD case. The coupling between the TCR/CD3 complex and the antigen peptide results in T-cell development, activation, proliferation, and cytokine production, including both T helper and T cytotoxic lymphocytes (75). The activation and differentiation of both cells are necessary for antibodies production (by T helper lymphocytes) and the intestinal epithelial damage (by T cytotoxic lymphocytes), leading to more lymphocyte infiltration and more immune response. The differentiation processes of both cells were enriched in CD3E and also for many GWAS genes in the PPIN. CD3E monoclonal antibodies have been utilized to modify the immune response, lower T-cell responsiveness to self-antigen, and treat autoimmune disorders (76–80). Several T-cell and thymus features are altered in CD3E gene KO/mutated mouse models (81, 82), and gene defects have also been associated with immunodeficiency and higher DM1 susceptibility in females (83). Epigenetic changes in genes involved in T cell signaling were found in Graves' disease patients including CD3E gene, as well as downregulation of other CDE genes (84). Finally, among Finish families with CeD and skin manifestations, the area 11q23 containing the CD3E chromosomal position—specifically the microsatellite marker D11S4142—demonstrated a substantial linkage maximum likelihood score (MLS). However, patients with only intestinal symptoms showed a lower MLS (85).

We sincerely acknowledge some limitations in this study. First, we studied only two celiac families, and studying more families could help us in validating the actual role of gene-specific rare variants in CeD. However, owing to the multilayered approach adopted in this study, our findings could act as proof of concept that RVB can help in dissecting the genetic complexity of CeD, where classical Mendelian segregation models are of limited value. Second, the absence of CeD GWAS data from the Arab population of the Middle East could create confounding bias in the disease risk assessment and misclassify the actual disease causative alleles. However, to control this ethnic-specific allele bias, we removed the common variants of the Arab population by comparing our exome data with the local population genetic database (SHGP and GME).

## Conclusion

This study highlights the genetic heterogeneity of CeD and necessitates the need for novel approaches to dissect its complex molecular basis. The present investigation lays forth a multidimensional approach to integrate the WES, GWAS, and functional biology data for identifying CeD candidate genes. Our findings propose that the rare variants in two potential candidate genes (*IL1R1* and *CD3E*) identified in this study are likely to contribute to gluten insensitivity and CeD pathogenesis by modulating the T-cell selection and maturation, cytokine signaling, and adaptive immune response pathways. These findings also underscore the relevance of family-specific rare variant analysis in prioritizing the disease candidate genes; however, future studies need to assess whether our findings can be generalized to sporadic CeD cases. Moreover, multi-omics-based *in vitro* and *in vivo* investigations are also expected to validate the biological role of celiac candidate genes at the transcriptome, proteome, and metabolome levels.

## Data Availability Statement

The datasets presented in this article are not readily available because (a) participants' refusal to store or distribute the genomic data in the public domain and (b) as per the local Institutional Ethics Committee approval and national policy on genomic data sharing in the public domain outside the country. Allowed data under the above mentioned restrictions of the IRB and participants requirements is presented in the article as well in the Supplementary Files, further inquiries can be directed to the corresponding author/s.

## Ethics Statement

The studies involving human participants were reviewed and approved by Research Ethics Committee, King Abdulaziz University Hospital, Jeddah (KAUH). Written informed consent to participate in this study was provided by the participants' legal guardian/next of kin.

## Author Contributions

BB, RE, and NS: conceptualization. HM, BB, and RE: methodology. HM and BB: software and visualization. HM, BB, and KN: formal analysis. HM, OS, BB, RE, and NS: investigation. BB and NS: resources. HM, NS, and RE: writing—original draft preparation. BB, RE, NS, OS, and JA: writing—review and editing. NS, BB, and RE: supervision. NS: project administration and funding acquisition. All authors contributed to the article and approved the submitted version.

## Funding

This project was funded by the National Plan for Science, Technology and Innovation (MAARIFAH), King Abdulaziz City for Science and Technology, the Kingdom of Saudi Arabia, Award Number 13-MED2225-03.

## Conflict of Interest

The authors declare that the research was conducted in the absence of any commercial or financial relationships that could be construed as a potential conflict of interest.

## Publisher's Note

All claims expressed in this article are solely those of the authors and do not necessarily represent those of their affiliated organizations, or those of the publisher, the editors and the reviewers. Any product that may be evaluated in this article, or claim that may be made by its manufacturer, is not guaranteed or endorsed by the publisher.

## References

[1] Singh P Arora A Strand TA Leffler DA Catassi C Green PH. Global prevalence of celiac disease: systematic review and meta-analysis. Clin Gastroenterol Hepatol. (2018) 16:823–836.e822. 10.1016/j.cgh.2017.06.037 29551598

[2] Aljebreen AM Almadi MA Alhammad A Al Faleh FZ. Seroprevalence of celiac disease among healthy adolescents in Saudi Arabia. World J Gastroenterol. (2013) 19:2374–8. 10.3748/wjg.v19.i15.2374 23613632 PMC3631990

[3] Constantin C Huber WD Granditsch G Weghofer M Valenta R. Different profiles of wheat antigens are recognised by patients suffering from coeliac disease and IgE-mediated food allergy. Int Arch Allergy Immunol. (2005) 138:257–66. 10.1159/000088727 16215327

[4] Subramaniam G Girish M. Iron deficiency anemia in children. Indian J Pediatr. (2015) 82:558–64. 10.1007/s12098-014-1643-9 25636824

[5] Martin-Masot R Nestares MT Diaz-Castro J Lopez-Aliaga I Alferez MJM Moreno-Fernandez J. Multifactorial etiology of anemia in celiac disease and effect of gluten-free diet: a comprehensive review. Nutrients. (2019) 11:2557. 10.3390/nu11112557 31652803 PMC6893537

[6] Ludvigsson JF Leffler DA Bai JC Biagi F Fasano A Green PH. The Oslo definitions for coeliac disease and related terms. Gut. (2013) 62:43–52. 10.1136/gutjnl-2011-301346 22345659 PMC3440559

[7] Mahadev S Murray JA Wu TT Chandan VS Torbenson MS Kelly CP. Factors associated with villus atrophy in symptomatic coeliac disease patients on a gluten-free diet. Aliment Pharmacol Ther. (2017) 45:1084–93. 10.1111/apt.13988 28220520

[8] Leffler DA Dennis M Hyett B Kelly E Schuppan D Kelly CP. Etiologies and predictors of diagnosis in nonresponsive celiac disease. Clin Gastroenterol Hepatol. (2007) 5:445–50. 10.1016/j.cgh.2006.12.006 17382600

[9] Schumann M Lebenheim L. [Celiac disease]. Dtsch Med Wochenschr. (2016) 141:1474–7. 10.1055/s-0042-112099 27701694

[10] Lewis D Haridy J Newnham ED. Testing for coeliac disease. Austr Prescriber. (2017) 40:105–8. 10.18773/austprescr.2017.029 28798516 PMC5478399

[11] Singh P Arora S Lal S Strand TA Makharia GK. Risk of celiac disease in the first- and second-degree relatives of patients with celiac disease: a systematic review and meta-analysis. Am J Gastroenterol. (2015) 110:1539–48. 10.1038/ajg.2015.296 26416192

[12] Hummel M Bonifacio E Stern M Dittler J Schimmel A Ziegler AG. Development of celiac disease-associated antibodies in offspring of parents with type I diabetes. Diabetologia. (2000) 43:1005–11. 10.1007/s001250051483 10990078

[13] Pham-Short A Donaghue KC Ambler G Phelan H Twigg S Craig ME. Screening for celiac disease in type 1 diabetes: a systematic review. Pediatrics. (2015) 136:e170–6. 10.1542/peds.2014-2883 26077482

[14] Bartoloni E Bistoni O Alunno A Cavagna L Nalotto L Baldini C. Celiac disease prevalence is increased in primary sjogren's syndrome and diffuse systemic sclerosis: lessons from a large multi-center study. J Clin Med. (2019) 8:40540. 10.3390/jcm8040540 31010199 PMC6517955

[15] Ferrari SM Fallahi P Ruffilli I Elia G Ragusa F Benvenga S. The association of other autoimmune diseases in patients with Graves' disease (with or without ophthalmopathy): Review of the literature and report of a large series. Autoimmun Rev. (2019) 18:287–92. 10.1016/j.autrev.2018.10.001 30639646

[16] Glissen Brown JR Singh P. Coeliac disease. Paediatr Int Child Health. (2019) 39:23–31. 10.1080/20469047.2018.1504431 30099930

[17] Gutierrez-Achury J Zhernakova A Pulit SL Trynka G Hunt KA Romanos J. Fine mapping in the MHC region accounts for 18% additional genetic risk for celiac disease. Nat Genet. (2015) 47:577–8. 10.1038/ng.3268 25894500 PMC4449296

[18] Lindfors K Ciacci C Kurppa K Lundin KEA Makharia GK Mearin ML. Coeliac disease. Nat Rev Dis Primers. (2019) 5:3. 10.1038/s41572-018-0054-z 30631077

[19] Liu E Rewers M Eisenbarth GS. Genetic testing: who should do the testing and what is the role of genetic testing in the setting of celiac disease? Gastroenterology. (2005) 128:S33–7. 10.1053/j.gastro.2005.02.013 15825124

[20] Welter D Macarthur J Morales J Burdett T Hall P Junkins H. The NHGRI GWAS Catalog, a curated resource of SNP-trait associations. Nucleic Acids Res. (2014) 42:D1001–6. 10.1093/nar/gkt1229 24316577 PMC3965119

[21] Trynka G Hunt KA Bockett NA Romanos J Mistry V Szperl A. Dense genotyping identifies and localizes multiple common and rare variant association signals in celiac disease. Nat Genet. (2011) 43:1193–201. 10.1038/ng.998 22057235 PMC3242065

[22] Van Heel DA Franke L Hunt KA Gwilliam R Zhernakova A Inouye M. A genome-wide association study for celiac disease identifies risk variants in the region harboring IL2 and IL21. Nat Genet. (2007) 39:827–9. 10.1038/ng2058 17558408 PMC2274985

[23] Hunt KA Zhernakova A Turner G Heap GA Franke L Bruinenberg M. Newly identified genetic risk variants for celiac disease related to the immune response. Nat Genet. (2008) 40:395–402. 10.1038/ng.102 18311140 PMC2673512

[24] Dubois PC Trynka G Franke L Hunt KA Romanos J Curtotti A. Multiple common variants for celiac disease influencing immune gene expression. Nat Genet. (2010) 42:295–302. 10.1038/ng.543 20190752 PMC2847618

[25] Saadah OI Shaik NA Banaganapalli B Salama MA Al-Harthi SE Wang J. Replication of GWAS coding SNPs implicates MMEL1 as a potential susceptibility locus among saudi arabian celiac disease patients. Dis Markers. (2015) 2015:351673. 10.1155/2015/351673 26843707 PMC4710944

[26] Szperl AM Ricaño-Ponce I Li JK Deelen P Kanterakis A Plagnol V. Exome sequencing in a family segregating for celiac disease. Clin Genet. (2011) 80:138–47. 10.1111/j.1399-0004.2011.01714.x 21627641

[27] Bokhari HA Shaik NA Banaganapalli B Nasser KK Ageel HI Al Shamrani AS. Whole exome sequencing of a Saudi family and systems biology analysis identifies CPED1 as a putative causative gene to Celiac Disease. Saudi J Biol Sci. (2020) 27:1494–502. 10.1016/j.sjbs.2020.04.011 32489286 PMC7254030

[28] Al-Aama JY Shaik NA Banaganapalli B Salama MA Rashidi O Sahly AN. Whole exome sequencing of a consanguineous family identifies the possible modifying effect of a globally rare AK5 allelic variant in celiac disease development among Saudi patients. PLoS ONE. (2017) 12:e0176664. 10.1371/journal.pone.0176664 28505210 PMC5432167

[29] Banaganapalli B Mansour H Mohammed A Alharthi AM Aljuaid NM Nasser KK. Exploring celiac disease candidate pathways by global gene expression profiling and gene network cluster analysis. Sci Rep. (2020) 10:16290. 10.1038/s41598-020-73288-6 33004927 PMC7529771

[30] Khalkhal E Nobakht F Haidari MH Razaghi Z Ghasemzad M Sheikhan M. Evaluation of expression of common genes in the intestine and peripheral blood mononuclear cells (PBMC) associated with celiac disease. Gastroenterol Hepatol Bed Bench. (2020) 13:S60–7. 33585005 PMC7881404

[31] Bragde H Jansson U Jarlsfelt I Söderman J. Gene expression profiling of duodenal biopsies discriminates celiac disease mucosa from normal mucosa. Pediatr Res. (2011) 69:530–7. 10.1203/PDR.0b013e318217ecec 21378598

[32] Khalkhal E Razzaghi Z Zali H Bahadorimonfared A Iranshahi M Rostami-Nejad M. Comparison of cytokine and gene activities in tissue and blood samples of patients with celiac disease. Gastroenterol Hepatol Bed Bench. (2019) 12:S108–16. 32099610 PMC7011060

[33] Leonard MM Bai Y Serena G Nickerson KP Camhi S Sturgeon C. RNA sequencing of intestinal mucosa reveals novel pathways functionally linked to celiac disease pathogenesis. PLoS ONE. (2019) 14:e0215132. 10.1371/journal.pone.0215132 30998704 PMC6472737

[34] Garner CP Murray JA Ding YC Tien Z Van Heel DA Neuhausen SL. Replication of celiac disease UK genome-wide association study results in a US population. Hum Mol Genet. (2009) 18:4219–25. 10.1093/hmg/ddp364 19648293 PMC2758145

[35] Mistry V Bockett NA Levine AP Mirza MM Hunt KA Ciclitira PJ. Exome sequencing of 75 individuals from multiply affected coeliac families and large scale resequencing follow up. PLoS ONE. (2015) 10:e0116845. 10.1371/journal.pone.0116845 25635822 PMC4312029

[36] Romanos J Barisani D Trynka G Zhernakova A Bardella MT Wijmenga C. Six new coeliac disease loci replicated in an Italian population confirm association with coeliac disease. J Med Genet. (2009) 46:60–3. 10.1136/jmg.2008.061457 18805825

[37] Plaza-Izurieta L Castellanos-Rubio A Irastorza I Fernández-Jimenez N Gutierrez G Bilbao JR. Revisiting genome wide association studies (GWAS) in coeliac disease: replication study in Spanish population and expression analysis of candidate genes. J Med Genet. (2011) 48:493–6. 10.1136/jmg.2011.089714 21490378

[38] Wu Y Gettler K Giri M Li D Bayrak CS Jain A. Identifying novel high-impact rare disease-causing mutations, genes and pathways in exomes of Ashkenazi Jewish inflammatory bowel disease patients. medRxiv. (2020) 2020.2007.2001.20143750. 10.1016/S0016-5085(20)32653-6

[39] Backman JD Li AH Marcketta A Sun D Mbatchou J Kessler MD. Exome sequencing and analysis of 454,787 UK Biobank participants. Nature. (2021) 599:628–34. 10.1038/s41586-021-04103-z 34662886 PMC8596853

[40] Husby S Koletzko S Korponay-Szab ó IR Mearin ML Phillips A Shamir R. European society for pediatric gastroenterology, hepatology, and nutrition guidelines for the diagnosis of coeliac disease. J Pediatr Gastroenterol Nutr. (2012) 54:136–60. 10.1097/MPG.0b013e31821a23d0 22197856

[41] Li H Handsaker B Wysoker A Fennell T Ruan J Homer N. The sequence alignment/map format and SAMtools. Bioinformatics. (2009) 25:2078–9. 10.1093/bioinformatics/btp352 19505943 PMC2723002

[42] Basha M Demeer B Revencu N Helaers R Theys S Bou Saba S. Whole exome sequencing identifies mutations in 10% of patients with familial non-syndromic cleft lip and/or palate in genes mutated in well-known syndromes. J Med Genet. (2018) 55:449. 10.1136/jmedgenet-2017-105110 29500247

[43] Wang K Li M Hakonarson H. ANNOVAR: functional annotation of genetic variants from high-throughput sequencing data. Nucleic Acids Res. (2010) 38:e164. 10.1093/nar/gkq603 20601685 PMC2938201

[44] Yang H Wang K. Genomic variant annotation and prioritization with ANNOVAR and wANNOVAR. Nat Protoc. (2015) 10:1556–66. 10.1038/nprot.2015.105 26379229 PMC4718734

[45] Ye J Coulouris G Zaretskaya I Cutcutache I Rozen S Madden TL. Primer-BLAST: A tool to design target-specific primers for polymerase chain reaction. BMC Bioinformatics. (2012) 13:134. 10.1186/1471-2105-13-134 22708584 PMC3412702

[46] Ajabnoor GMA Mohammed NA Banaganapalli B Abdullah LS Bondagji ON Mansouri N. Expanded somatic mutation spectrum of MED12 Gene in uterine leiomyomas of Saudi Arabian women. Front Genet. (2018) 9:552. 10.3389/fgene.2018.00552 30619444 PMC6302612

[47] Elsokary HA Abdullah LS Ujaimi A Sahly NN Mansouri N Banaganapalli B. Assessing the role of serum prolactin levels and coding region somatic mutations of the prolactin gene in Saudi uterine leiomyoma patients. Arch Med Sci. (2020). 10.5114/aoms.2020.98658 PMC12892275 41684567

[48] Banaganapalli B Rashidi O Saadah OI Wang J Khan IA Al-Aama JY. Comprehensive computational analysis of GWAS loci identifies CCR2 as a candidate gene for celiac disease pathogenesis. J Cell Biochem. (2017) 118:2193–207. 10.1002/jcb.25864 28059456

[49] Shannon P Markiel A Ozier O Baliga NS Wang JT Ramage D. Cytoscape: a software environment for integrated models of biomolecular interaction networks. Genome Res. (2003) 13:2498–504. 10.1101/gr.1239303 14597658 PMC403769

[50] Bindea G Mlecnik B Hackl H Charoentong P Tosolini M Kirilovsky A. ClueGO: a Cytoscape plug-in to decipher functionally grouped gene ontology and pathway annotation networks. Bioinformatics. (2009) 25:1091–3. 10.1093/bioinformatics/btp101 19237447 PMC2666812

[51] Bindea G Galon J Mlecnik B. CluePedia Cytoscape plugin: pathway insights using integrated experimental and in silico data. Bioinformatics. (2013) 29:661–3. 10.1093/bioinformatics/btt019 23325622 PMC3582273

[52] Assenov Y Ramírez F Schelhorn SE Lengauer T Albrecht M. Computing topological parameters of biological networks. Bioinformatics. (2008) 24:282–4. 10.1093/bioinformatics/btm554 18006545

[53] Bult CJ Blake JA Smith CL Kadin JA Richardson JE. Mouse genome database (MGD) 2019. Nucleic Acids Res. (2019) 47:D801–6. 10.1093/nar/gky1056 30407599 PMC6323923

[54] Mclaren W Gil L Hunt SE Riat HS Ritchie GRS Thormann A. The ensembl variant effect predictor. Genome Biol. (2016) 17:122. 10.1186/s13059-016-0974-4 27268795 PMC4893825

[55] Mistry J Chuguransky S Williams L Qureshi M Salazar Gustavo a. Pfam: The protein families database in 2021. Nucleic Acids Res. (2021) 49:D412–9. 10.1093/nar/gkaa913 33125078 PMC7779014

[56] Altschul SF Madden TL Schäffer AA Zhang J Zhang Z Miller W. Gapped BLAST and PSI-BLAST: a new generation of protein database search programs. Nucleic Acids Res. (1997) 25:3389–402. 10.1093/nar/25.17.3389 9254694 PMC146917

[57] Berman HM Westbrook J Feng Z Gilliland G Bhat TN Weissig H. The protein data bank. Nucleic Acids Res. (2000) 28:235–42. 10.1093/nar/28.1.235 10592235 PMC102472

[58] Shen MY Sali A. Statistical potential for assessment and prediction of protein structures. Protein Sci. (2006) 15:2507–24. 10.1110/ps.062416606 17075131 PMC2242414

[59] Laskowski RA MacArthur MW Moss DS Thornton JM. it PROCHECK: a program to check the stereochemical quality of protein structures. J Appl Crystallography. (1993) 26:283–91. 10.1107/S0021889892009944

[60] Pires DE Ascher DB Blundell TL. DUET: a server for predicting effects of mutations on protein stability using an integrated computational approach. Nucleic Acids Res. (2014) 42:W314–9. 10.1093/nar/gku411 24829462 PMC4086143

[61] Janson G Zhang C Prado MG Paiardini A. PyMod 2.0: improvements in protein sequence-structure analysis and homology modeling within PyMOL. Bioinformatics. (2017) 33:444–6. 10.1093/bioinformatics/btw638 28158668

[62] Schlessinger A Yachdav G Rost B. PROFbval: predict flexible and rigid residues in proteins. Bioinformatics. (2006) 22:891–3. 10.1093/bioinformatics/btl032 16455751

[63] Feng YCA Howrigan DP Abbott LE Tashman K Cerrato F Singh T. Ultra-rare genetic variation in the epilepsies: a whole-exome sequencing study of 17,606 individuals. Am J Human Genetics. (2019) 105:267–82. 10.1016/j.ajhg.2019.05.020 31327507 PMC6698801

[64] Balicza P Varga NÁ Bolgár B Pentelényi K Bencsik R Gál A. Comprehensive analysis of rare variants of 101 autism-linked genes in a Hungarian cohort of autism spectrum disorder patients. Front Genet. (2019) 10:434. 10.3389/fgene.2019.00434 31134136 PMC6517558

[65] Halvorsen M Huh R Oskolkov N Wen J Netotea S Giusti-Rodriguez P. Increased burden of ultra-rare structural variants localizing to boundaries of topologically associated domains in schizophrenia. Nat Commun. (2020) 11:1842. 10.1038/s41467-020-15707-w 32296054 PMC7160146

[66] Naruse H Ishiura H Mitsui J Takahashi Y Matsukawa T Tanaka M. Burden of rare variants in causative genes for amyotrophic lateral sclerosis (ALS) accelerates age at onset of ALS. J Neurol. (2019) 90:537. 10.1136/jnnp-2018-318568 30355605

[67] Mountjoy E Schmidt EM Carmona M Schwartzentruber J Peat G Miranda A. An open approach to systematically prioritize causal variants and genes at all published human GWAS trait-associated loci. Nat Genet. (2021) 53:1527–33. 10.1038/s41588-021-00945-5 34711957 PMC7611956

[68] Fields JK Günther S Sundberg EJ. Structural basis of IL-1 family cytokine signaling. Front Immunol. (2019) 10:e01412. 10.3389/fimmu.2019.01412 31281320 PMC6596353

[69] Dinarello CA. Immunological and inflammatory functions of the interleukin-1 family. Annu Rev Immunol. (2009) 27:519–50. 10.1146/annurev.immunol.021908.132612 19302047

[70] Manavalan JS Hernandez L Shah JG Konikkara J Naiyer AJ Lee AR. Serum cytokine elevations in celiac disease: association with disease presentation. Hum Immunol. (2010) 71:50–7. 10.1016/j.humimm.2009.09.351 19735687

[71] Fornari MC Pedreira S Niveloni S González D Diez RA Vázquez H. Pre- and post-treatment serum levels of cytokines IL-1beta, IL-6, and IL-1 receptor antagonist in celiac disease. Are they related to the associated osteopenia? Am J Gastroenterol. (1998) 93:413–8. 10.1111/j.1572-0241.1998.00413.x 9580142

[72] Nasserinejad M Shojaee S Ghobakhlou M Lak E Eslami P Pourhoseingholi MA. The effects of IL-8, IL- 6, and IL-1 on the risk of celiac disease: a Bayesian regression analysis. Gastroenterol Hepatol Bed Bench. (2019) 12:S117–22. 32099611 PMC7011063

[73] Palová-Jelínková L Dánová K Drašarová H Dvorák M Funda DP Fundová P. Pepsin digest of wheat gliadin fraction increases production of IL-1β via TLR4/MyD88/TRIF/MAPK/NF-κB signaling pathway and an NLRP3 inflammasome activation. PLoS ONE. (2013) 8:e62426. 10.1371/journal.pone.0062426 23658628 PMC3639175

[74] Harris KM Fasano A Mann DL. Cutting edge: IL-1 controls the IL-23 response induced by gliadin, the etiologic agent in celiac disease. J Immunol. (2008) 181:4457–60. 10.4049/jimmunol.181.7.4457 18802048

[75] Chatenoud L. CD3-specific antibodies restore self-tolerance: mechanisms and clinical applications. Curr Opin Immunol. (2005) 17:632–7. 10.1016/j.coi.2005.09.011 16214320

[76] Kuhns MS Davis MM Garcia KC. Deconstructing the form and function of the TCR/CD3 complex. Immunity. (2006) 24:133–9. 10.1016/j.immuni.2006.01.006 16473826

[77] Gaglia J Kissler S. Anti-CD3 antibody for the prevention of type 1 diabetes: a story of perseverance. Biochemistry. (2019) 58:4107–11. 10.1021/acs.biochem.9b00707 31523950 PMC6918689

[78] Sugita S Shimizu J Makabe K Keino H Watanabe T Takahashi M. Inhibition of T cell-mediated inflammation in uveitis by a novel anti-CD3 antibody. Arthritis Res Ther. (2017) 19:176. 10.1186/s13075-017-1379-9 28743289 PMC5526238

[79] Munder M Bettelli E Monney L Slavik JM Nicholson LB Kuchroo VK. Reduced self-reactivity of an autoreactive T cell after activation with cross-reactive non-self-ligand. J Exp Med. (2002) 196:1151–62. 10.1084/jem.20020390 12417626 PMC2194103

[80] Marrella V Poliani PL Fontana E Casati A Maina V Cassani B. Anti-CD3ε mAb improves thymic architecture and prevents autoimmune manifestations in a mouse model of Omenn syndrome: therapeutic implications. Blood. (2012) 120:1005–14. 10.1182/blood-2012-01-406827 22723555 PMC3470012

[81] Malissen M Gillet A Ardouin L Bouvier G Trucy J Ferrier P. Altered T cell development in mice with a targeted mutation of the CD3-epsilon gene. Embo j. (1995) 14:4641–53. 10.1002/j.1460-2075.1995.tb00146.x 7588594 PMC394561

[82] Sommers CL Dejarnette JB Huang K Lee J El-Khoury D Shores EW. Function of CD3 epsilon-mediated signals in T cell development. J Exp Med. (2000) 192:913–9. 10.1084/jem.192.6.913 10993922 PMC2193290

[83] Wong S Moore S Orisio S Millward A Demaine AG. Susceptibility to type I diabetes in women is associated with the CD3 epsilon locus on chromosome 11. Clin Exp Immunol. (1991) 83:69–73. 10.1111/j.1365-2249.1991.tb05590.x 1671006 PMC1535466

[84] Limbach M Saare M Tserel L Kisand K Eglit T Sauer S. Epigenetic profiling in CD4+ and CD8+ T cells from Graves' disease patients reveals changes in genes associated with T cell receptor signaling. J Autoimmun. (2016) 67:46–56. 10.1016/j.jaut.2015.09.006 26459776

[85] Holopainen P Mustalahti K Uimari P Collin P Mäki M Partanen J. Candidate gene regions and genetic heterogeneity in gluten sensitivity. Gut. (2001) 48:696–701. 10.1136/gut.48.5.696 11302971 PMC1728294

