# Supplementary material for: Genome-Wide Association Study-Guided Exome Rare Variant Burden Analysis Identifies IL1R1 and CD3E as Potential Autoimmunity Risk Genes for Celiac Disease
Source: Front Pediatr. 2022 Feb 14;10:837957. doi: 10.3389/fped.2022.837957 (PMC8882628; doi:10.3389/fped.2022.837957)
Supplement: Supplementary file 1 [file Data_Sheet_1.docx]

**Supplementary figure S1:** Sanger sequencing results of IGFN1 autosomal recessive variant of family A (c.3056T>G). Sequencing results show heterozygous genotype (TG) in the parents, affected-3 and normal-3. Homozygous wild type (TT) is seen in normal-1 and normal-2. Homozygous mutated type (GG) is seen in affected-1, affected-2, and normal-4.

**Supplementary figure S2:** Sanger sequencing results of LAD1 autosomal recessive variant of family A (c.452G>A). Sequencing results show heterozygous genotype (GA) in the parents, affected-2, affected-3, and normal-3. Homozygous wild type (GG) is seen in normal-1 and normal-2. Homozygous mutated type (AA) is seen in affected-1 and normal-4.

**
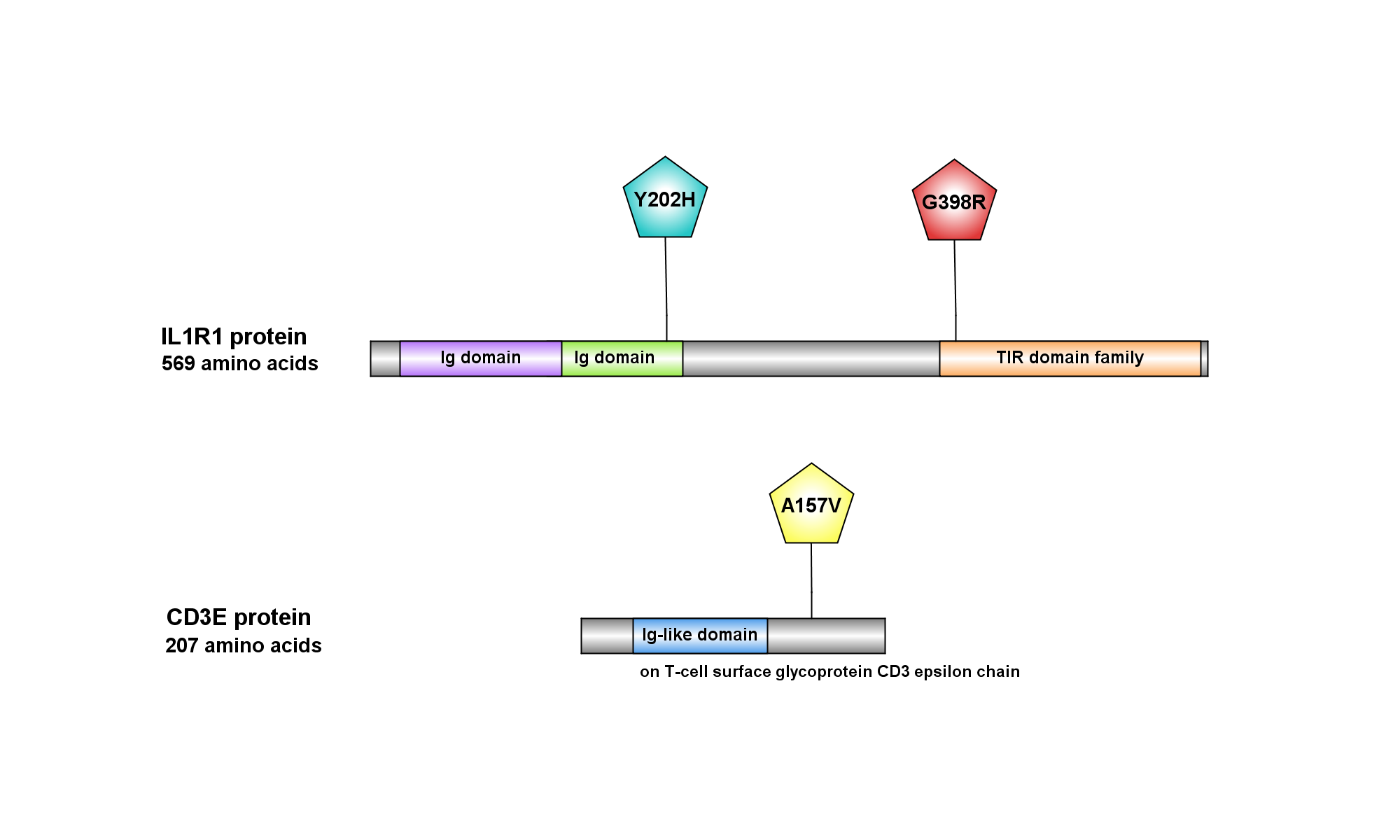
**

**Suppimentary Figure S3:**The position of IL1R1 and CD3E variants relative to protein domain regions. (A): both Y202H and G398R Il1R1 variants are mapped to the domain regions immunoglobulin domain and Toll-Interleukin receptor, respectivly. (B) However, CD3E’s A157V variant was not.

Supplementary Figure S4: Computational validation of buildup protein structures of IL1R1 (A) and CD3E (B) using PROCHECK program, more than 97% residues are in favored and allowed regions.
