# Supplementary material for: Genome-Wide Association Study-Guided Exome Rare Variant Burden Analysis Identifies IL1R1 and CD3E as Potential Autoimmunity Risk Genes for Celiac Disease
Source: Front Pediatr. 2022 Feb 14;10:837957. doi: 10.3389/fped.2022.837957 (PMC8882628; doi:10.3389/fped.2022.837957)

**Supplementary Table S1:** The inclusion and exclusion criteria followed for filtering WES data.

| **Selection criteria** | **Included variants** | **Excluded variants** |
| --- | --- | --- |
| Variant minor allele frequency (MAF) | Low MAF (< 0.02) | High MAF (> 0.02) |
| Variant depth read | > 30 | < 30 |
| Variant maximum quality read (MQ) | > 40 | < 40 |
| Alternative depth to total depth ratio | 40-70% for heterozygous variants  > 80% for homozygous variants | < 40% or > 70% for heterozygous variants  < 80% for homozygous variants |
| Variant effect | Coding variants:   - In frame and frame shift deletion and insertion - Splice site variants (donor or acceptor) - Missense variants - Stop and start loss and gain variants - Structural interaction variants | Non-coding and regulatory variants:   - 3’ and 5’ UTR variants - Conservative in frame and frame shift deletion and insertion - Intron variants - Synonymous variants - Initiator codon variants |

Supplementary Table S2: Polymerase chain reaction conditions followed for amplifying target gene regions.

| **Step** | **Tm** | **Duration** | **No.of cycles** |
| --- | --- | --- | --- |
| Initial denaturation | 95^0^C | 3 minutes | 1 |
| Denaturation | 95^0^C | 30 seconds | 35 |
| Annealing | 58^0^C | 30 seconds |  |
| Extension | 72^0^C | 30 seconds |  |
| Final extension | 72^0^C | 7 minutes | 1 |
| Hold | 4^0^C | - | - |

|  | Family A | | | | Family B | | | |
| --- | --- | --- | --- | --- | --- | --- | --- | --- |
|  | Father | Affected 1 | Affected 2 | Normal | Father | Mother | Affected 1 | Affected 2 |
| All variants | 75486 | 74531 | 75547 | 77696 | 103197 | 103197 | 105220 | 105896 |
| Rare variants (MAF < 0.02) | 10759 | 10031 | 10117 | 11350 | 20506 | 21160 | 21278 | 21335 |
| QC filterd variants | 5306 | 4952 | 4823 | 5450 | 10758 | 11269 | 14883 | 11842 |
| Coding variants | 1129 | 1035 | 1009 | 1119 | 1766 | 1827 | 2809 | 1847 |
| Homozygous variants | 134 | 165 | 133 | 126 | 169 | 151 | 83 | 83 |
| Heterozygous variants | 788 | 672 | 719 | 780 | 777 | 749 | 731 | 659 |

**Supplementary Table S3:** The distribution of whole exome sequencing generated variants for families A and B against different filtering options.

**Supplementary Table S4**: Innate immunity genes mapped from exome sequencing datasets of celiac disease patients.

| **Innate immunity genes**  **found in Family A**  **(21 genes)** | **Innate immunity genes**  **found in Family B**  **(13 genes)** |
| --- | --- |
| C9 | ASCC3 |
| CLEC4M | C1QA |
| CXCR1 | DUOX2 |
| IL1R1 | EHMT2 |
| IL1RL1 | FPR2 |
| KLRG1 | HLA-B |
| MAP3K1 | HLA-DQB1 |
| MMP8 | KLRC2 |
| MX1 | LAIR1 |
| NOD1 | MAP2K3 |
| PLEC | NLRP8 |
| RARRES2 | PLEC |
| SCN5A | ZFPM1 |
| SERPINE1 |  |
| SIGLEC6 |  |
| SLX4 |  |
| TNRC6B |  |
| TRIM42 |  |
| TRIM67 |  |
| TRPM2 |  |
| TUFM |  |

Supplementary Table S5: The functional enrichment of celiac disease hub genes highlights the GO terms and pathways related to the immune system**.**


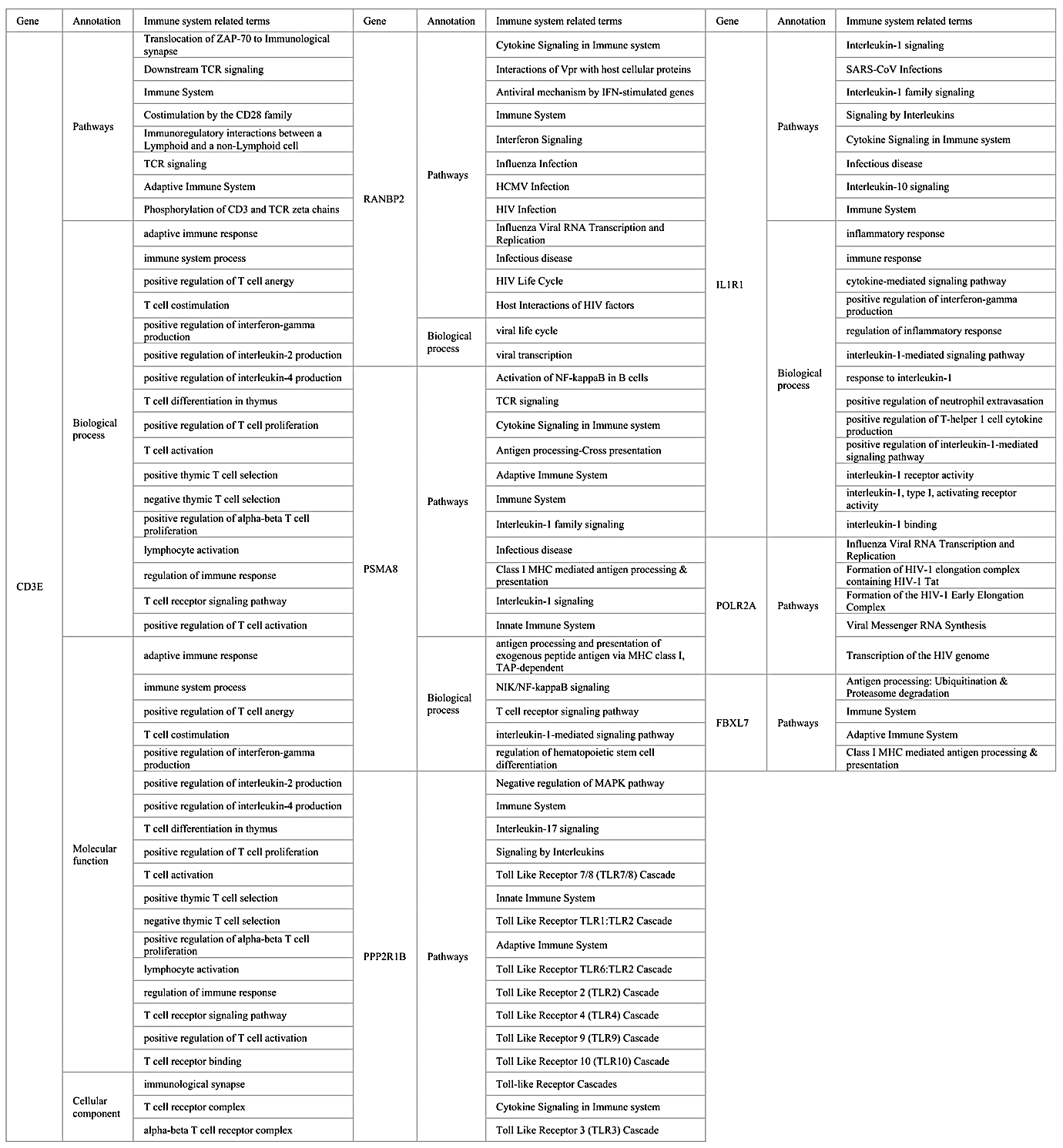

Supplement: Supplementary file 2 [file Data_Sheet_2.docx]
